# The Color Improvement of Underwater Images Based on Light Source and Detector

**DOI:** 10.3390/s22020692

**Published:** 2022-01-17

**Authors:** Xiangqian Quan, Yucong Wei, Bo Li, Kaibin Liu, Chen Li, Bing Zhang, Jingchuan Yang

**Affiliations:** 1 Institute of Deep-Sea Science and Engineering, Chinese Academy of Sciences, Sanya 572000, China; weiyucong@idsse.ac.cn (Y.W.); libo@idsse.ac.cn (B.L.); liukb@idsse.ac.cn (K.L.); lic@idsse.ac.cn (C.L.); zhangb@idsse.ac.cn (B.Z.); yangjc@idsse.ac.cn (J.Y.); 2 University of Chinese Academy of Sciences, Beijing 100049, China

**Keywords:** underwater optics, color improvement, underwater imaging

## Abstract

As one of the most direct approaches to perceive the world, optical images can provide plenty of useful information for underwater applications. However, underwater images often present color deviation due to the light attenuation in the water, which reduces the efficiency and accuracy in underwater applications. To improve the color reproduction of underwater images, we proposed a method with adjusting the spectral component of the light source and the spectral response of the detector. Then, we built the experimental setup to study the color deviation of underwater images with different lamps and different cameras. The experimental results showed that, a) in terms of light source, the color deviation of an underwater image with warm light LED (Light Emitting Diode) (with the value of $\sqrt{\Delta a{*}^{2}+\Delta b{*}^{2}}$ being 26.58) was the smallest compared with other lamps, b) in terms of detectors, the color deviation of images with the 3×CMOS RGB camera (a novel underwater camera with three CMOS sensors developed for suppressing the color deviation in our team) (with the value of $\sqrt{\Delta a{*}^{2}+\Delta b{*}^{2}}$ being 25.25) was the smallest compared with other cameras. The experimental result (i.e., the result of color improvement between different lamps or between different cameras) verified our assumption that the underwater image color could be improved by adjusting the spectral component of the light source and the spectral response of the detector. Differing from the color improvement method with image processing, this color-improvement method was based on hardware, which had advantages, including more image information being retained and less-time being consumed.

## 1. Introduction

### 1.1. The Background of Color Improvement in Marine Surveys

As one of the most direct approaches to perceive the world, optical images can provide plenty of useful information for various underwater applications, such as marine geology surveys, underwater mining, fishery, and marine archaeology [1,2,3]. However, differing from the optical imaging systems in the terrestrial environment, underwater imaging systems generally suffer from color deviation due to the varying degrees of attenuation encountered by light traveling in water with different wavelengths. The color deviation impacts the reliability and utility in underwater applications [4,5]. There is no doubt that the color improvement of underwater images is significant for underwater applications [6,7,8,9], and the scope of color improvement in underwater images has received considerable attention in recent decades [10,11]. To solve the color deviation of the underwater image, lots of research has been conducted, which can be classified into two categories, including color restoration with prior information of light attenuation, and color enhancement without the information of light attenuation.

### 1.2. Related Work

#### 1.2.1. Color Restoration with the Prior Information of Light Attenuation

Color restoration with prior information is a method based on the analysis of light attenuation. This method requires three steps, including analyzing attenuation characteristics of light in water, building an underwater imaging model, and restoring the color of the underwater image by data processing [12,13]. First, the attenuation of light could be analyzed in terms of spectrum or color channels [14]. In [15], Kan et al. considered the nonlinear attenuation of light in different wavelengths at different depths, then the change in three color channel values was calculated and used to compensate for the color loss. In [16], after analyzing the influence of the spectral discretization on an underwater image, the color reproduction of the underwater images was studied by Boffety. In [17], to calculate the attenuation in water, Kaeli et al. proposed a novel method to estimate the attenuation coefficient with a Doppler velocity log. Second, an underwater imaging model was built. One general model of the underwater imaging process is the Jaffe–McGlamery model, where the irradiance of a monochromatic underwater image is formulated as the linear combination of the following three components: the direct component, the absorption component, and the scattering component [18,19]. In our team, to tackle the color deviation caused by artificial lighting, we proposed a new model of underwater image degradation, where the parameters of deep-sea lamps were added [20]. In [21], Guo et al. presented a model representing the absorption, scattering, and refraction of water, lenses, and image sensors. In [22], Lu et al. proposed a novel underwater imaging model to compensate for the attenuation discrepancy along the propagation path. In [23], Eunpil Park et al. proposed a novel underwater image-formation model in which forward scattering was included. Lastly, the color was compensated by data processing. The Dark Channel Prior (DCP) from outdoor image dehazing was introduced in underwater image color restoration [24]. In [25], Galdran et al. proposed the Red Channel Prior based on the DCP to recover the lost contrast in underwater images. This new prior reversed the red channel to deal with the strong attenuation of red light in water bodies. In [26], Drews Jr et al. proposed the Underwater DCP (UDCP) from the traditional DCP by excluding the red channel used in producing the prior. Apart from the DCP-related priors, there are also other priors proposed for underwater image restoration. In [27], Meng et al. proposed a hybrid method for the color-improvement process based on a principle that exploited the relationship of R, G, and B (red, green, and blue) channels. Validation proved that the method had a better performance. Codruta et al. introduced a single-image approach, which built on the blending of two images directly derived from a color-compensated and white-balanced board of the original degraded image. The evaluation revealed that the enhanced images and videos were characterized by improved color reproduction [28].

#### 1.2.2. Color Enhancement without the Information of Light Attenuation

Color enhancement without the information of light attenuation is based on data processing, which does not require specialized hardware or knowledge about underwater conditions or scene structure [29]. This method could be divided into three categories, including the Retinex algorithm, Contrast Limited Adaptive Histogram Equalization algorithm (CLAHE), and deep learning algorithm. In terms of the Retinex algorithm, Hassan performed a Retinex-based enhancement of a CLAHE-processed image; the qualitative and quantitative performance comparison with some of the existing approaches showed that the proposed approach achieved better enhancement of the underwater images [30]. In [31], Tang et al. proposed a new underwater image-enhancement algorithm based on adaptive feedback and the Retinex algorithm; the result showed that the color saturation, color richness, and clarity of the image were all significantly improved. Jobson et al. extended a previously designed single-scale center/surround Retinex to a multi-scale version that achieved simultaneous dynamic range compression/color consistency/lightness rendition [32]. Shu Zhang et al. presented a novel method, namely LAB-MSR, achieved by modifying the original Retinex algorithm. It utilized the combination of the bilateral filter and trilateral filter on the three channels of the image in CIELAB color space according to the characteristics of each channel [33]. In terms of CLAHE, Iqbal et al. used histogram stretching in the RGB color space to restore the color balance. The saturation and intensity stretching of HSI was used to increase the true color and solve the problem of lighting [34]. In [35], Ahmad et al. integrated the modification of image histogram into two main color models consisting of Red–Green–Blue and Hue–Saturation–Value color spaces. Qualitative analysis revealed that the proposed method could significantly reduce the blue–green effect. In terms of a deep learning algorithm, to reduce the amount of data required while providing better image enhancement, Deng et al. proposes an underwater image color transfer generative adversarial network (UCT-GAN) [36]. In [37], Chen et al. proposed a new underwater image enhancement method based on deep learning and an image formation model. In [38], Lu et al. proposed a multi-scale cycle generative adversarial network system including the information of structural similarity index measure loss, dark channel prior algorithm, and adaptive structural similarity index measure loss. A strong performance on the underwater image color correction was acquired. Furthermore, many synthesis algorithms have been proposed. In [39], Kamil et al. proposed a natural method based on an underwater image color enhancement method consisting of four steps, including introducing a new approach to neutralize underwater the color cast, proposing dual-intensity images fusion based on the average of mean and median values, proposing swarm intelligence based on mean equalization, and applying the unsharp masking technique. Experiments on underwater images captured under various conditions indicated that the proposed method could improve the image color reproduction significantly. In [40], Li et al. proposed a weakly supervised color transfer method to correct color deviation by designing a multi-term loss function, including adversarial loss, cycle consistency loss, and structural similarity index measure loss. The experiments showed that the method produced visually pleasing results.

### 1.3. Our Work

Despite the above color improvement methods having advantages in cost and robustness, the methods also have caused the loss of information and the consumption of time. There is no doubt that these methods go against the rapid judgment and intelligent recognition in underwater applications. To address these problems, we proposed a color-improvement method with adjusting the spectral component of light source and the spectral response of detector. To verify our assumption, first, the underwater imaging model was analyzed. Then, an experiment platform for analyzing the color deviation of underwater optical imaging was designed, and the color deviation of underwater images with different lamps and different cameras was acquired. The experimental results showed that the color deviation of an underwater image with a warm-light LED (Light-Emitting Diode) (with a value of $\sqrt{\Delta a{*}^{2}+\Delta b{*}^{2}}$ being 26.58) is the smallest compared to other lamps, and the color deviation of an image with the 3×CMOS RGB camera (a novel underwater camera with three CMOS sensors developed in our team for suppressing the color deviation (with the value of $\sqrt{\Delta a{*}^{2}+\Delta b{*}^{2}}$ being 25.25)) is the smallest compared to other cameras. The result verified our assumption of color improvement by adjusting the spectral component of light source and the spectral response of detector. Furthermore, differing from the color-improvement method with image processing, this color-improvement method was based on hardware, which has advantages, including more image information being retained and less time being consumed, which are significant in the rapid judgment and real-time video transmission of underwater applications.

## 2. Experimental Setup and Details

### 2.1. The Analysis of the Underwater Imaging Process

**Figure 1:**
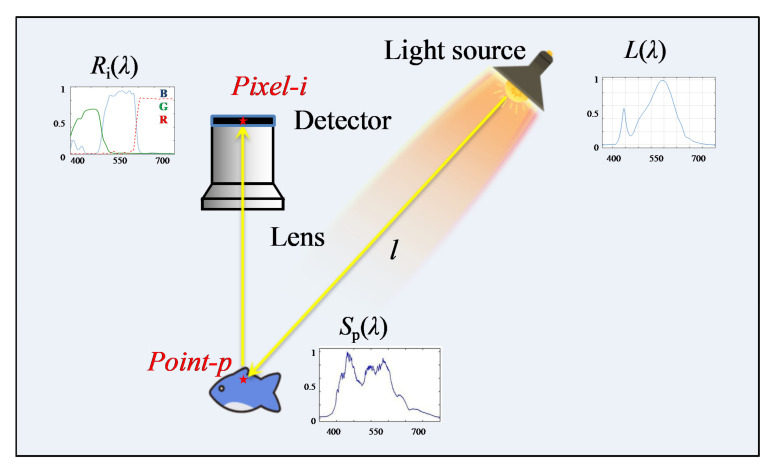
The sketch map of the underwater imaging process.

The underwater imaging process is shown in Figure 1, wherein the light from the light source penetrates through the water to the target. Then, the light is reflected by the object to the lens and detector; the point p is imaged to the pixel i in detector. $L\left(\lambda \right)$ denotes the spectral component of the light source, $S_{p}\left(\lambda \right)$ is the reflection function of point p at wavelength λ, and $R_{i}\left(\lambda \right)$ denotes the spectral response of pixel i at wavelength λ.

In the underwater imaging model, the intensity values of a certain pixel i of the detector from point p, $V_{i,p}$, can be calculated as

(1) $$V_{i,p}=\int_{\lambda }L\left(\lambda \right)S_{p}\left(\lambda \right)R_{i}\left(\lambda \right)e^{-\mu \left(\lambda \right)l\left(p\right)}d\lambda$$

where $\mu \left(\lambda \right)$ denotes the light attenuation coefficient at wavelength λ. $l\left(p\right)$ denotes the optical path of attenuation from light source to point p to lens. As is shown in Equation (1), there are four factors that influence the intensity value $V_{i,p}$. The attenuation (i.e., $\mu \left(\lambda \right)$ and $l\left(p\right)$) is the root cause for color deviation. The reflectance ($S_{p}\left(\lambda \right)$) is an intrinsic feature of the object. The spectral component ($L\left(\lambda \right)$) of the lamp and the spectral response of detector ($R_{i}\left(\lambda \right)$) can be modified by us, so a method adjusting the spectral distribution performance of lighting source and the spectral response function of detector is proposed.

### 2.2. Experimental Setup and Process

**Figure 2:**
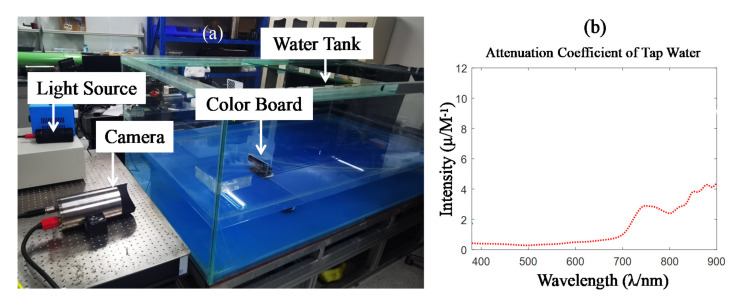
The experimental setup for color reproduction analysis of underwater images: (**a**) the experimental setup of capturing underwater images; (**b**) the attenuation coefficient of tap water.

To verify our assumption, the experimental setup was built as shown in Figure 2a. The camera and lamp were placed outside the water tank; the test target, i.e., the 24 color board, was put inside the water tank. The attenuation coefficient of water is shown in Figure 2b. The experiment was carried out in a dark room. The light penetrates through the water to the 24-color board and is reflected at the camera, with changes in the distance between the target to the wall of the tank (which is the imaging distance in water). The color reproduction of underwater images with different lamps and cameras can be derived. The distance parameter is just a variate for selecting samples, and our main conclusion is not relevant to the distance parameter. Therefore, we set the imaging distance from 0 m to 2 m.

**Figure 3:**
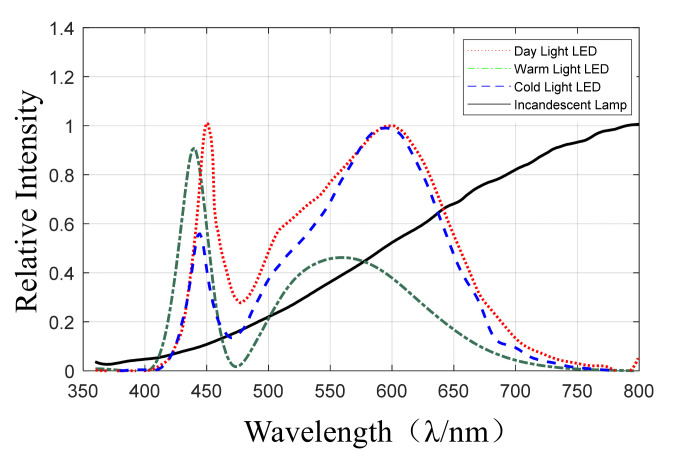
The spectral component curves of different lamps.

To analyze the effect of the light source on color reproduction of the underwater image, four kinds of lamps (including the day light LED, warm light LED, cold light LED, and incandescent lamp) were applied in the experiment. The spectral component curves of the four lamps are shown in Figure 3.

Meanwhile, to verify the effect of the detector on color improvement of underwater images, four cameras, including three consumer cameras were used. Camera No.1 is the camera of HUAWEI mobile phone LIO-AL00, in which the white balance is set to 5500 k, corresponding to the environment of indirect sunlight on sunny days; camera No.2 is a camera of Hikvision DS-2CD7087EWD-A, in which the white balance is set to indirect sunlight on sunny days (i.e., 5500 k); and camera No.3 is a GoPro hero7-1, in which the white balance is also set to 5500 k. The detailed specs of these three cameras are as follows:

The specifications of camera No.1—HUAWEI mobile phone LIO-AL00:

- The camera contains four sub-cameras with 3× optical zoom (with 18 mm, 27 mm, 80 mm) and 30× digital zoom;
- The resolution is 3840 × 2160;
- The white balance setting is 5500 k;
- The shutter speed is 1/125 s;
- The exposure compensation is 0;
- The ISO is 640;
- AF (auto focus) is AF-C.

The specifications of camera No.2—Hikvision DS-2CD7087EWD-A:

- The resolution is 3840 × 2160;
- The size of detector is 1/1.8”;
- The minimum illumination in color mode is 0.002 Lux@F1.2;
- The white balance setting is indirect sunlight on sunny days (i.e., 5500 k);
- The digital noise reduction level is set to 50;
- The brightness is set to 50;
- The contrast ratio is set to 50;
- The sharpness is set to 50;
- The saturation is set to 50;
- The shutter speed is 1/25 s;
- The day/night conversion mode is turned off;
- The Backlight compensation function is turned off.

The specifications of camera No.3—GoPro hero7-1:

- The resolution is 12 megapixels;
- The white balance setting is 5500 k;
- The sharpness setting is moderate;
- The shutter speed is 1/125 s;
- The exposure compensation is 0;
- ISO is set from 100 to 3200;
- The function of color is set to flat;
- The FOV is set to linearity;
- The function of super phone is turned off.

**Figure 4:**
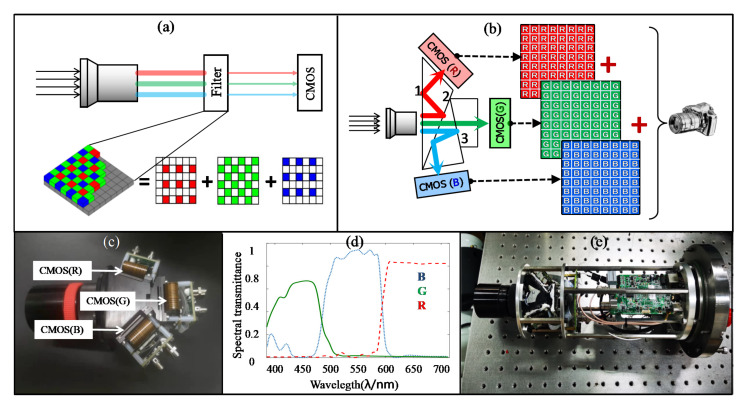
The design of the 3×CMOS RGB camera: (**a**) the imaging principle of traditional color; (**b**) the imaging principle of traditional of 3×CMOS RGB camera; (**c**) the photo of spectral structure with prism; (**d**) the transmittance curve of R, G, and B channels; (**e**) the photo of the 3×CMOS RGB camera movement.

The 3×CMOS RGB camera design of our team was the fourth camera applied in this experiment (Figure 4 shows the difference between the traditional camera and 3×CMOS RGB camera). Figure 4a shows the imaging principle of traditional color camera, where the filter consists of three types of micro filters corresponding R, G, and B channels. Figure 4b shows the imaging principle of 3×CMOS RGB camera, where the polychromatic light is split into R, G, B colors with the coating prism. Then, by composing data from three detectors corresponding to R, G, and B channels, the color image is derived. Figure 4c shows the photo of spectral structure with prism. Figure 4d shows the transmittance curve of R, G, and B channels, in which the transmittance of red channel is increased by coating. Figure 4e shows the photo of the 3×CMOS RGB camera movement. The improvement of underwater image color is presented between the 3×CMOS camera with the three cheap cameras (the “raw images” were captured by the three common camera, while the “improved images” were captured by 3×CMOS camera).

**Figure 5:**
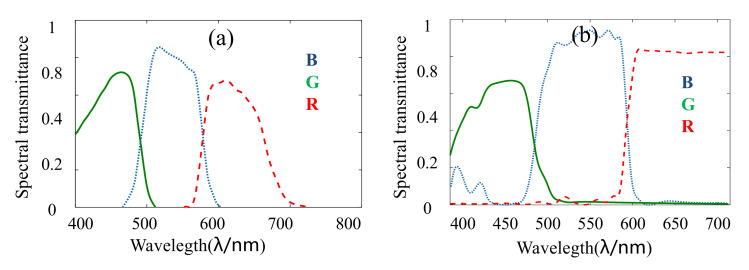
The difference in transmittance between common prism spectral structure and the newly designed prism spectral structure: (**a**) the spectral transmittance of common prism spectral structure; (**b**) the spectral transmittance of newly designed prism spectral structure.

In the 3×CMOS RGB camera, the coatings of the prism spectral structure are modified by increasing the transmittance of the red wave band. The difference in transmittance between common prism spectral structure with the newly designed prism spectral structure is shown in Figure 5. In the calibration of image color, the white balance algorithm is designed to retain the advantage of increasing the transmittance of the red wave band of the prism spectral structure.

**Figure 6:**
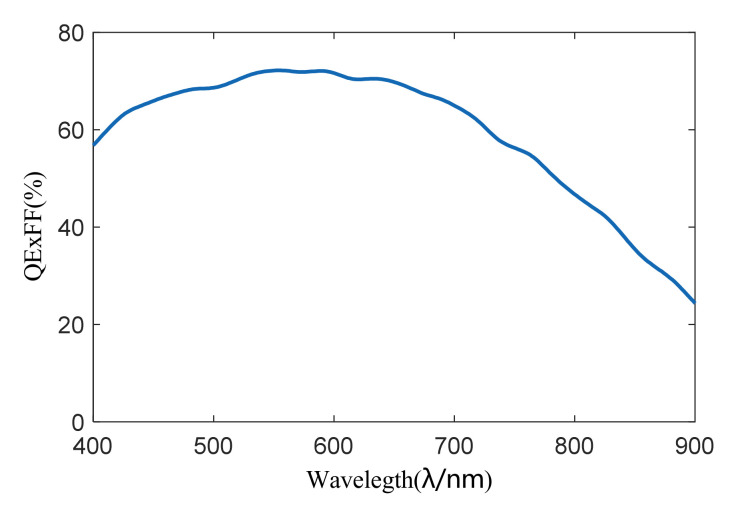
The spectral response of CMOS sensor GSENSE2011E.

The CMOS sensors are GSENSE2011E from Gpixel Inc. GSENSE2011E features 2e^−^ readout noise, 87.5dB intra-scene dynamic range, and frame rate up to 668fps. The number of pixels is 2048 (H) × 1152 (W), the pixel size is 6.5 μm × 6.5 μm, and the detector has an outstanding quantum efficiency of 72% at 595 nm. The spectral response of the detector is shown in Figure 6. 

The F number of the lens is 4. The working wavelength band is 400–700 nm. The lens is fixed focal with the focal distance is 12 mm. The resolution is 2048 × 1152. The minimum illumination is 0.003Lux@F4.0. The shutter speed is 1/60 s. The raw images are on based three channel CMOS sensors with Cameralink-based interface, the compressed output images are one channel HD-SDI images with data processing of FPGA.

**Figure 7:**
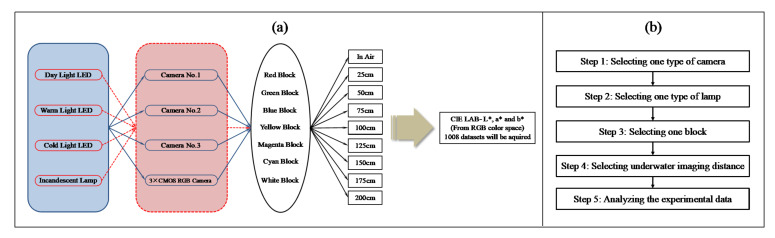
The experimental process: (**a**) the process of setting experimental parameters; (**b**) the flow chat of experimental process.

The experimental process is shown in Figure 7. By setting experimental parameters, including the light source, camera, test color block, and work distance, a dataset including the values of R, G, and B can be derived. Figure 7a shows the process of setting experimental parameters; Figure 7b shows the flow chart of experimental process.

**Figure 8:**
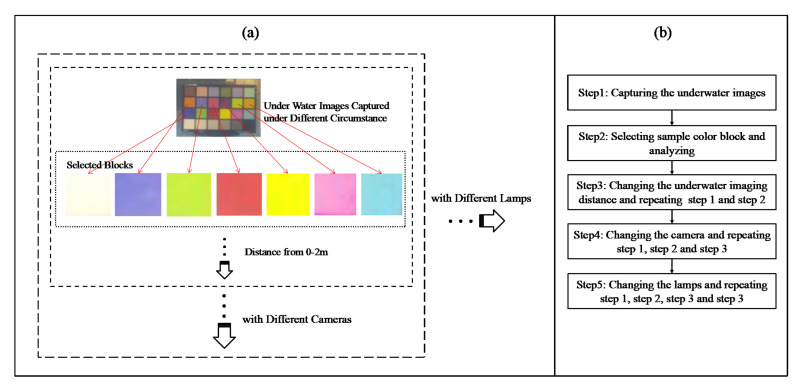
The process of acquiring experiment data: (**a**) the sketch map of data acquisition; (**b**) the flow chat of data acquisition.

The process of acquiring experiment data is shown in Figure 8. As is shown in Figure 8a, the underwater images were captured first; then, 7 color blocks were extracted. With the change in distance, lamps, and cameras, 144 images and 1008 blocks were captured. Figure 8b shows the acquisition process of this experiment images data. Lastly, the R, G, and B values of experiment images were acquired.

## 3. Experimental Results

Based on the acquisition process of experiment images data (shown as Figure 8), 144 color images and 1008 selected blocks were captured in the experiment. As it was difficult to show them, we turned the form of experiment results from image to data. To remove the effect of brightness on experiment images, the image data were converted from the RGB color space to the CIELAB color space according to Equations (2)–(4), where L* is the parameter to measure brightness (which is ignored in this paper), and a* and b* are the parameters to measure color [41].

(2) $$\left\{\begin{array}{l} L*=116\left(Y/Y_{0}\right){\;}^{1/3}-16\\ a*=500\left[f\left(X/0.9505\right)-f\left(Y\right)\right]\\ b*=200\left[f\left(Y\right)-f\left(Z/1.0891\right)\right] \end{array}\right.$$

where X, Y, Z are derived from Equation (3) and $f\left(t\right)$ is expressed as Equation (4). R, G, and B are the values of image data corresponding to R, G, and B channels.

(3) $$\left[\begin{array}{l} X\\ Y\\ Z \end{array}\right]=\left[\begin{array}{lll} 0.412453 & 0.357580 & 0.180423\\ 0.212671 & 0.715160 & 0.072169\\ 0.019334 & 0.119193 & 0.950227 \end{array}\right]\left[\begin{array}{l} R\\ G\\ B \end{array}\right]$$

(4) $$f\left(t\right)=\left\{\begin{array}{ll} t^{1/3} & \mathrm{if}\;t>{\left(\frac{6}{29}\right)}^{3}\\ \frac{1}{3}{\left(\frac{29}{6}\right)}^{2}t+\frac{4}{29} & \mathrm{otherwise} \end{array}\right.$$

**Figure 9:**
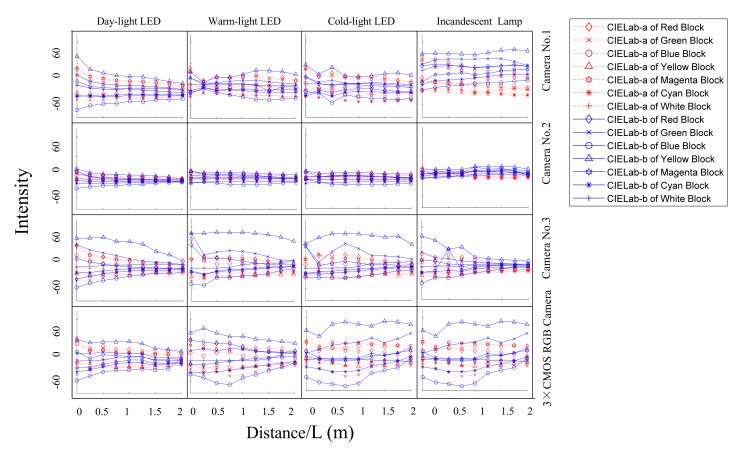
The values of a* and b* at different distances with different lamps and different cameras.

The values of a* and b* at different distances with four types of lamps and four types of cameras can be calculated as Figure 9.

**Figure 10:**
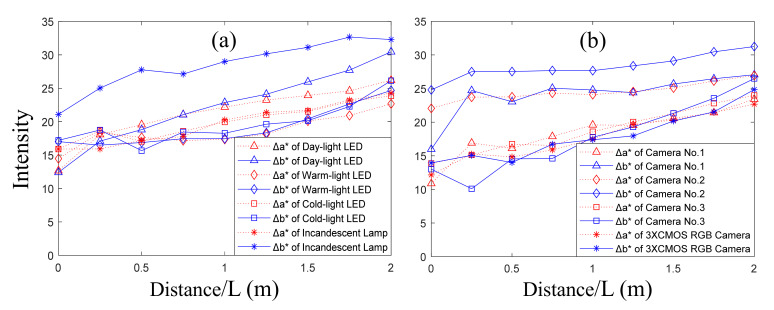
The values of Δa* and Δb* with four kinds of lamps and four kinds of cameras: (**a**) Δa* and Δb* with different lamps; (**b**) Δa* and Δb* with different cameras.

By calculating the difference in the values of a* and b* between experimental images and calibration images, the color deviation parameters Δa* and Δb* with different light lamps and different cameras are shown in Figure 10. It can be concluded that the color deviation increases with the increase in the imaging distance. 

Based on the data in Figure 10a, the color deviation can be derived as in Table 1. We can conclude that the color deviation of the underwater image with the warm-light LED is the smallest (the value of $\sqrt{\Delta a{*}^{2}+\Delta b{*}^{2}}$ is 26.58).

Based on the data in Figure 10b, the color deviation with different cameras can be calculated as in Table 2. We can conclude that the color deviation with the 3×CMOS RGB camera is the smallest (the value of $\sqrt{\Delta a{*}^{2}+\Delta b{*}^{2}}$ is 25.25).

## 4. Conclusions and Discussion

By analyzing light attenuation in water and the imaging model, we proposed a method to correct the color deviation by adjusting the spectral component of the light source and the spectral response of the detector. Then, an experimental setup for analyzing the color deviation in underwater images was set up, and the color deviation with different light sources and different cameras was analyzed quantitatively. The experiment’s results showed that the color reproduction of underwater images with a warm-light LED is superior to the other lamps and that the color deviation of underwater images with a 3×CMOS RGB camera, including adjusting the spectral response function of the detector, is superior to the other cameras.

## Figures and Tables

**Table 1:** The color deviation and nonlinear errors with different lamps.

Items		Day-Light LED	Warm-Light LED	Cold-Light LED	Incandescent Lamp
Δa*	In air	12.67	14.47	15.90	15.91
	2 m	26.20	22.67	23.87	24.27
	Mean value in water	21.28	18.54	19.95	19.74
Δb*	In air	12.41	17.02	17.20	21.09
	2 m	30.45	24.68	26.13	32.29
	Mean value in water	22.27	19.04	19.62	28.47
$\sqrt{\Delta a{*}^{2}+\Delta b{*}^{2}}$	In air	17.73	22.34	23.43	26.42
	2 m	40.17	33.51	35.40	40.40
	Mean value in water	30.80	26.58	27.98	34.65

**Table 2:** The Color deviation with different cameras.

Items		Camera No.1	Camera No.2	Camera No.3	3×CMOS RGB Camera
Δa*	In air	10.87	22.06	13.87	12.16
	2 m	23.42	26.94	23.99	22.68
	Mean value in water	18.48	24.52	18.77	17.75
Δb*	In air	15.97	24.80	13.00	13.94
	2 m	27.06	31.25	26.50	24.84
	Mean value in water	24.13	28.27	17.85	17.96
$\sqrt{\Delta a{*}^{2}+\Delta b{*}^{2}}$	In air	19.32	33.19	19.01	18.50
	2 m	35.79	41.26	35.74	32.82
	Mean value in water	30.39	37.41	25.91	25.25

## Data Availability

The data presented in this study are available from the corresponding author upon request.
